# Supplementary material for: Relationship Between Air Pollution and the Concentration of Nitric Oxide in the Exhaled Air (FeNO) in 8–9-Year-Old School Children in Krakow
Source: Int J Environ Res Public Health. 2021 Jun 22;18(13):6690. doi: 10.3390/ijerph18136690 (PMC8296872; doi:10.3390/ijerph18136690)
Supplement: Supplementary file 1 [file ijerph-18-06690-s001.zip › ijerph-1226873-supplementary.pdf]

International Journal of
Environmental Research and Public Health

MDPI

Supplementary Materials

Relationship between Air Pollution and the Concentration of Nitric Oxide in the Exhaled Air (FeNO) in 8-9-Year-Old School Children in KRAKOW

Marta Czubaj-Kowal, Ryszard Kurzawa, Henryk Mazurek, Michał Sokołowski, Teresa Friediger, Maciej Polakand Grzegorz Józef Nowicki

Table S1. Relationship between FeNO and air pollution in the study group after adjustment for age and sex.

**FeNO**	**Stage I (autumn-winter period):**			**Stage II (springer-summer period):**		
	b	SE	*p*	b	SE	*p*
The day of measurement:						
NO [μg/m³]	0.047	0.015	0.001	0.028	0.015	0.048
CO8h [μg/m³]	0.119	0.017	< 0.001	0.073	0.017	< 0.001
C_6H_6 [μg/m³]	0.109	0.019	< 0.001	0.011	0.015	0.45
PM_{10}[μg/m³]	0.089	0.015	< 0.001	0.025	0.015	0.08
$PM_{2.5}$[μg/m³]	0.090	0.015	< 0.001	0.036	0.015	0.01
The median value from the week before:						
NO [μg/m³]	0.028	0.014	0.049	0.029	0.013	0.04
CO8h [μg/m³]	0.051	0.017	0.003	0.033	0.016	0.04
C_6H_6 [μg/m³]	0.042	0.018	0.02	0.025	0.015	0.1
PM_{10}[μg/m³]	0.043	0.015	0.003	0.001	0.015	0.094
$PM_{2.5}$[μg/m³]	0.040	0.015	< 0.01	0.009	0.015	0.52

b: standardized beta coefficient; SE: standard error
